# Nationwide Survey of Institutional Factors Related to the Use of Gonadotropin‐Releasing Hormone Analogs for Ovarian Protection in Women Receiving Chemotherapy in Japan

**DOI:** 10.1111/jog.70273

**Published:** 2026-04-06

**Authors:** Shina Sakaguchi, Eishin Nakamura, Haipeng Huang, Yasushi Takai

**Affiliations:** ^1^ Center for Maternal, Fetal and Neonatal Medicine, Saitama Medical Center Saitama Medical University Saitama Japan; ^2^ Department of Obstetrics and Gynecology, Saitama Medical Center Saitama Medical University Saitama Japan; ^3^ Center for Regenerative Medicine National Center for Child Health and Development Tokyo Japan

**Keywords:** fertility preservation, gonadotropin‐releasing hormone analog, Japan, ovarian function, surveys and questionnaires

## Abstract

**Aim:**

This study aimed to investigate institutional factors associated with the clinical implementation of gonadotropin‐releasing hormone (GnRH) analogs for ovarian protection in women receiving chemotherapy among fertility preservation facilities in Japan.

**Methods:**

We conducted a nationwide, cross‐sectional, web‐based survey between May 1 and May 31, 2025, targeting all 172 medical facilities accredited by the Japan Society of Obstetrics and Gynecology (JSOG) as fertility preservation service providers. The questionnaire assessed institutional characteristics, awareness of the recommendation of GnRH agonists for ovarian protection, and current clinical use of GnRH analogs. Logistic regression analyses were performed to identify institutional factors associated with the use of GnRH agonists.

**Results:**

A total of 128 facilities responded (response rate, 74.4%). Although 83.6% of facilities were aware of the recommendation of GnRH agonists for ovarian protection, only 26.6% reported experience using them. University hospitals (adjusted odds ratio [aOR], 7.6; 95% confidence interval [CI], 3.0–20.6; *p* < 0.001) and facilities with a full‐time “oncofertility navigator” (aOR, 3.9; 95% CI, 1.3–13.6; *p* = 0.02) were more likely to report GnRH agonist use. Only 3.9% of facilities reported experience prescribing GnRH antagonists for ovarian protection, mainly in specific clinical situations such as urgent chemotherapy initiation or contraindications to GnRH agonists. Notably, 67.2% of responding facilities indicated that they would be more likely to use GnRH analogs for ovarian protection under the hypothetical condition that public insurance coverage were available.

**Conclusion:**

Despite high awareness of recommendations for GnRH agonists, the clinical implementation of GnRH analogs for ovarian protection remains limited in Japan. Institutional characteristics and policy‐related factors were associated with reported GnRH agonist use, although causal relationships cannot be inferred. These findings highlight a gap between current clinical practice and available evidence and underscore the need for careful evaluation of the role of GnRH analogs within existing fertility preservation frameworks.

## 1 Introduction

Chemotherapy and radiotherapy for children, adolescents, and young adult (CAYA) female patients with cancer carry a substantial risk of ovarian dysfunction and infertility [1]. With recent advances in cancer therapy, long‐term survival among CAYA cancer patients has markedly improved [2]; however, these cytotoxic treatments often damage the ovarian reserve, leading to premature ovarian insufficiency (POI) and loss of fertility potential [1]. As survival outcomes improve, preserving fertility and hormonal function has become an essential component of comprehensive cancer care, closely linked to patients' long‐term quality of life (QOL) [3, 4].

Gonadotropin‐releasing hormone (GnRH) analogs have been proposed as pharmacologic agents for ovarian protection in women receiving chemotherapy. In Japan, GnRH agonists such as leuprorelin acetate and GnRH antagonists such as relugolix are clinically available. By suppressing the hypothalamic–pituitary–ovarian axis, GnRH agonists induce a temporary hypo‐gonadotropic state that reduces follicular recruitment and maintains primordial follicles in a quiescent state [3, 4]. Experimental data have also suggested additional protective mechanisms, including decreased ovarian blood flow and anti‐apoptotic effects mediated through locally expressed GnRH receptors [5, 6, 7]. Likewise, GnRH antagonists, which directly block GnRH receptors and rapidly inhibit gonadotropin secretion, have demonstrated ovarian‐protective effects in preclinical animal models [8, 9, 10].

Despite these biologic rationales, the clinical evidence supporting the use of GnRH analogs for ovarian protection remains limited [11, 12]. In Japan, the Clinical Practice Guidelines for Fertility Preservation published by the Japan Society of Clinical Oncology (JSCO) conditionally recommend GnRH agonists for ovarian protection only as an alternative when fertility preservation is not pursued, and only for patients receiving chemotherapy for selected malignancies (e.g., breast cancer) [13]. Against this clinical positioning, the implementation of GnRH agonists appears to be influenced by differences in institutional infrastructure and the availability of specialized personnel, and international studies have reported considerable variability in clinical practice across institutions and countries [14, 15, 16, 17, 18, 19, 20, 21]. However, in Japan, where fertility preservation medicine has been rapidly evolving, the extent to which GnRH analogs are used for ovarian protection and the institutional factors influencing their implementation have not been sufficiently clarified.

Therefore, this study aimed to investigate awareness of recommendations for GnRH agonists and the current clinical use of GnRH analogs in Japan, and to identify institutional factors associated with their implementation, providing foundational information on real‐world clinical practice for future discussions on fertility preservation strategies.

## 2 Methods

### 2.1 Study Design and Setting

This was a nationwide, cross‐sectional, web‐based survey conducted in Japan between May 1 and May 31, 2025. The study aimed to investigate institutional factors associated with the clinical use of GnRH analogs for ovarian protection during chemotherapy. The survey targeted all 172 medical facilities accredited by the Japan Society of Obstetrics and Gynecology (JSOG) as fertility preservation service providers.

### 2.2 Participants

Among the 172 JSOG‐accredited fertility preservation facilities, one individual responsible for fertility preservation at each institution was invited to participate. Participation was voluntary, and only one response per facility was accepted. Completion of the questionnaire was considered to indicate consent to participate.

### 2.3 Data Collection and Variables

Data were collected using an online questionnaire created with Google Forms. The questionnaire was distributed to eligible JSOG‐accredited fertility preservation facilities and completed electronically by respondents. It included both multiple‐choice and open‐ended questions, and the estimated completion time was approximately 5–10 min.

The questionnaire comprised items in three domains: (1) Facility characteristics, including annual volume of fertility preservation counseling and the presence of an institutional oncofertility support system; (2) clinical practices related to ovarian protection, including experience with GnRH agonists or antagonists used for ovarian protection, target malignancies, and cost categories (in this survey, the use of GnRH analogs was limited to ovarian protection, regardless of whether fertility preservation (e.g., oocyte or embryo cryopreservation) was performed, and use for cancer treatment purposes was excluded); and (3) institutional awareness and laboratory practices, including awareness of the Clinical Practice Guidelines for Fertility Preservation in Childhood, Adolescent and Young Adult Cancer Patients 2024 (JSCO Guidelines 2024) and the measurement of reproductive hormones—anti‐Müllerian hormone (AMH), follicle‐stimulating hormone (FSH), and estradiol—before and/or after chemotherapy. These items were included as indicators of institutional awareness of fertility preservation and routine clinical practice related to the assessment of ovarian reserve.

The complete questionnaire items are provided in Table S1. The primary outcome was the self‐reported use of GnRH analogs for ovarian protection within the respondent's own institution. The questionnaire was designed to explore awareness of GnRH analog use and its clinical implementation in real‐world practice.

### 2.4 Statistical Analysis

Descriptive statistics were used to summarize facility characteristics and survey responses. Categorical variables were presented as frequencies and percentages, and continuous variables were summarized as medians and interquartile ranges (IQRs) where appropriate. Missing data were excluded from analyses.

Univariable logistic regression analyses were first performed to examine associations between institutional characteristics and the use of GnRH agonists. Variables considered clinically important based on prior literature and expert opinion were then entered into a multivariable logistic regression model to identify independent factors associated with GnRH agonist use. Odds ratios (ORs) and 95% confidence intervals (CIs) were calculated. Model fit was evaluated using the likelihood ratio test and the Hosmer–Lemeshow goodness‐of‐fit test. All analyses were conducted using JMP Student Edition version 18.0 (SAS Institute Inc., Cary, NC, USA) and R version 4.5.1 (R Foundation for Statistical Computing, Vienna, Austria).

### 2.5 Ethical Considerations

The first question of the survey asked respondents to confirm their consent to participate, and only those who selected “Agree” were able to proceed to subsequent questions. All responses were collected anonymously, and no personal or facility‐identifiable information was obtained.

This study was approved by the Ethics Committee of Saitama Medical Center, Saitama Medical University (approval number: 2024‐232). It was conducted as part of the Ministry of Health, Labour and Welfare–sponsored research project “Establishment of psychosocial support systems for pediatric and AYA cancer patients in oncofertility care and development of a safe long‐term specimen storage system” (Research No. 23EA1016, Principal Investigator: Nao Suzuki).

## 3 Results

### 3.1 Facility Characteristics

**FIGURE 1:**
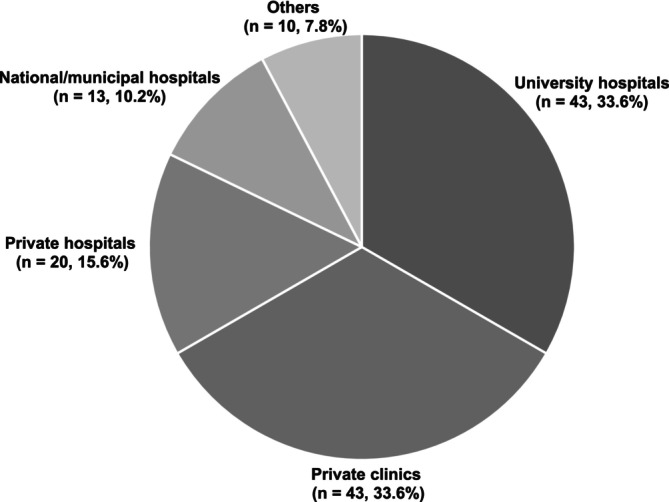
Distribution of fertility preservation facilities (*n* = 128). Values indicate the number of institutions (*n*) and the percentage of all responding institutions.

**FIGURE 2:**
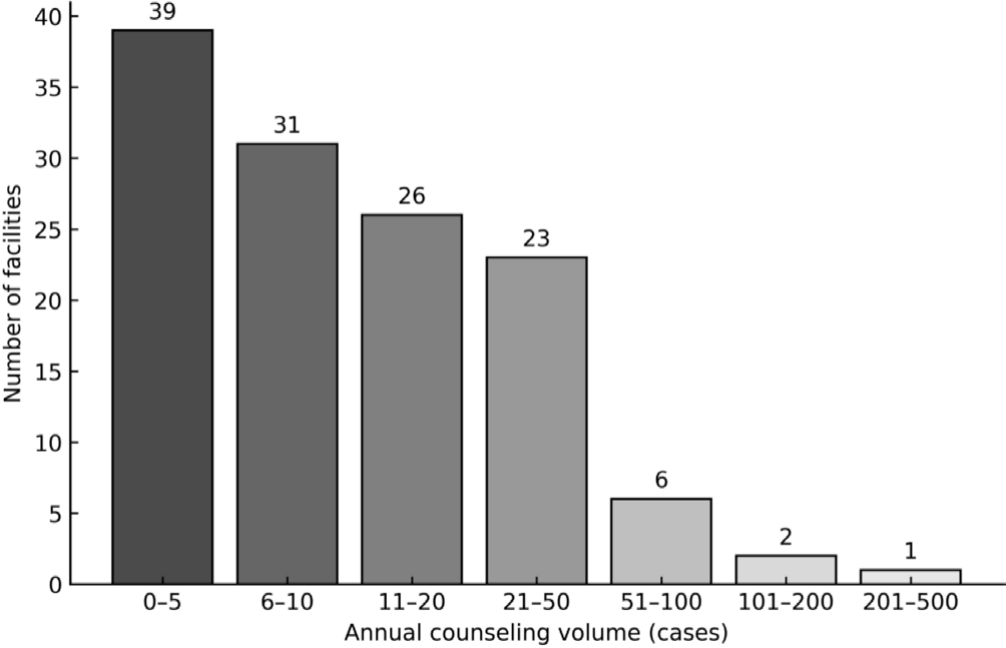
Annual number of fertility preservation counseling sessions per institution (*n* = 128). Most institutions reported fewer than 20 counseling sessions per year, with 0–5 sessions being the most common category (39 institutions, 30.5%).

**FIGURE 3:**
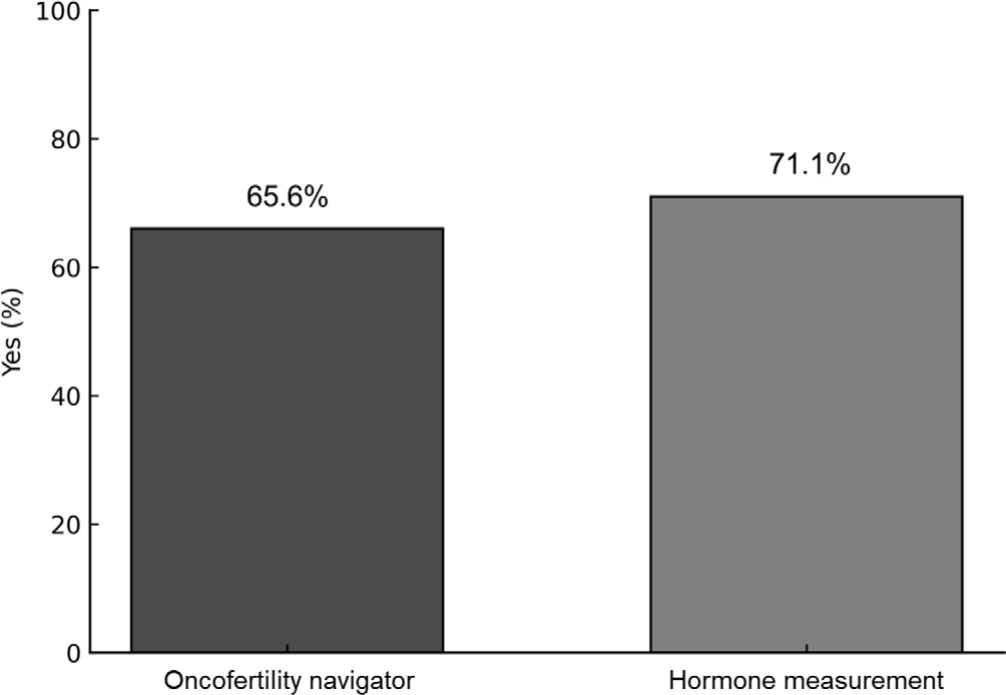
Presence of a full‐time “oncofertility navigator” and measurement of reproductive hormone levels before and/or after chemotherapy (*n* = 128). Among the responding institutions, 65.6% had a full‐time “oncofertility navigator,” and 71.1% measured reproductive hormone levels (anti‐Müllerian hormone [AMH], follicle‐stimulating hormone [FSH], and estradiol) before and/or after chemotherapy.

A total of 128 facilities responded to the survey among the 172 fertility preservation institutions invited nationwide (response rate, 74.4%). All responding facilities agreed to participate in the survey, and none declined participation. The distribution of facility types was as follows: university hospitals (43 institutions, 33.6%), private clinics (43, 33.6%), private hospitals (20, 15.6%), national/municipal hospitals (13, 10.2%), and others (10, 7.8%) (Figure 1). The annual number of counseling sessions showed a right‐skewed distribution, characterized by a small number of high‐volume facilities, with an overall median (IQR) of 10 (5–21) sessions. Ninety‐six facilities (75.0%) reported fewer than 20 fertility preservation counseling sessions per year. Of these, 39 facilities (30.5%) reported 0–5 sessions and 31 facilities (24.2%) reported 6–10 sessions (Figure 2). Among the respondents, 84 facilities (65.6%) reported having a full‐time “oncofertility navigator,” and 91 (71.1%) routinely measured reproductive hormone levels—including AMH, FSH, and estradiol—before or after chemotherapy (Figure 3).

### 3.2 Awareness and Experience With GnRH Agonists

**FIGURE 4:**
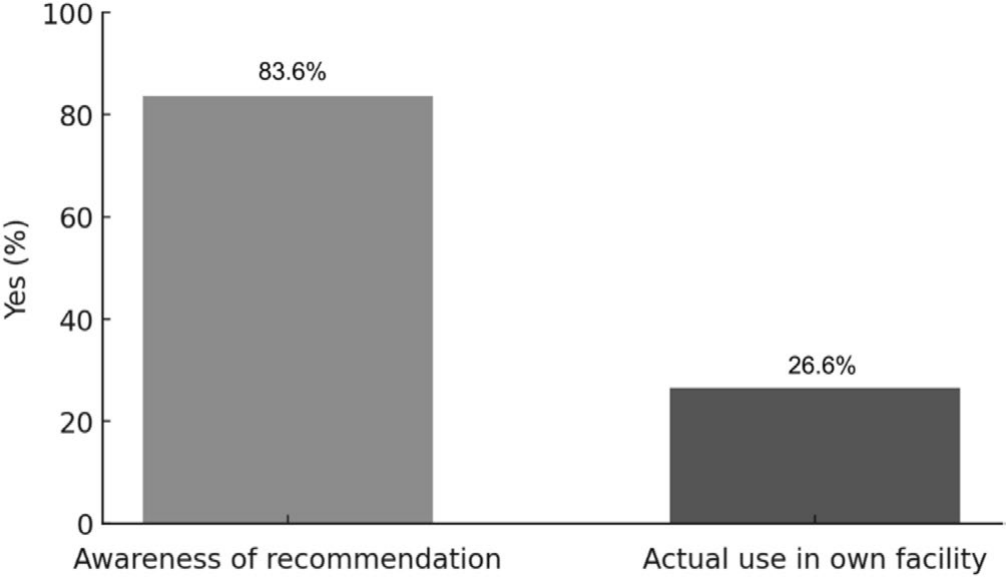
Awareness and actual use of GnRH agonists for ovarian protection (*n* = 128). While 83.6% of institutions were aware of the recommendation to use GnRH agonists for ovarian protection, only 26.6% had used them for this purpose in their own institution. Seven facilities (5.5%) responded “unknown.”

**FIGURE 5:**
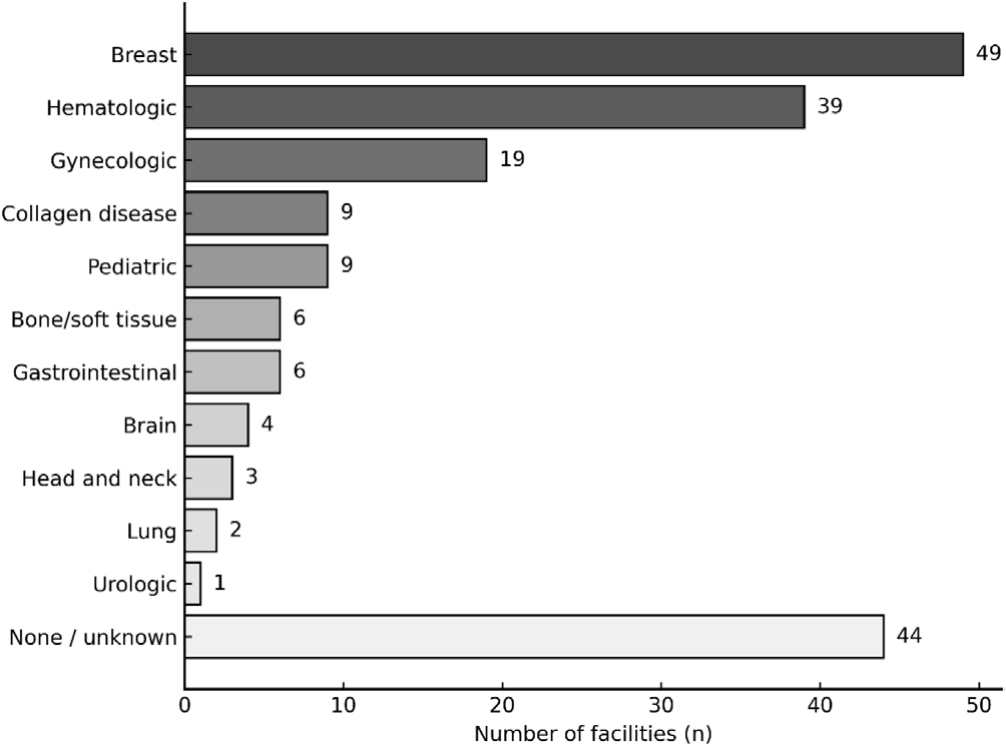
Malignancies for which GnRH agonists were selected for ovarian protection, including both cases used within the respondent's own institution and those encountered at other institutions (*n* = 114). Multiple responses were allowed. Numbers represent the total number of institutions selecting each indication. Breast cancer (49 institutions) and hematologic malignancies (39 institutions) were the most frequent indications, followed by gynecologic malignancies (19 institutions).

Among the 128 facilities, 107 (83.6%) were aware of the recommendation to use GnRH agonists for ovarian protection. Thirty‐four facilities (26.6%) reported having used GnRH agonists for ovarian protection in their own institutions, whereas 87 (68.0%) had no prior experience. Seven facilities (5.5%) responded “unknown” (Figure 4). The diseases for which GnRH agonists were selected for ovarian protection, including both use within the respondent's own institution and cases encountered at other institutions, are shown in Figure 5. The three most frequent were breast cancer (49 institutions, 43.0%), hematologic malignancies (39 institutions, 34.2%), and gynecologic malignancies (19 institutions, 16.7%), while 44 institutions (38.6%) responded “none/unknown.” Regarding cost coverage, 34 facilities responded, of which 10 (29.4%) reported out‐of‐pocket payment, 7 (20.5%) were covered by insurance, 15 (44.1%) were case‐dependent, and 2 (5.9%) were uncertain.

We examined factors influencing the experience with the use of GnRH agonists for ovarian protection using univariate logistic regression analysis. University hospital status (OR 7.74, 95% CI 3.31–19.13, *p* < 0.001), the presence of a full‐time “oncofertility navigator” (OR 4.11, 95% CI 1.57–12.92, *p* = 0.01), and awareness of the JSCO Guidelines 2024 (OR 3.44, 95% CI 1.31–10.83, *p* = 0.02) were significantly associated with this outcome. In contrast, awareness of the 2027 revision of facility certification criteria, measurement of AMH and reproductive hormones before and/or after chemotherapy, and the annual number of fertility preservation counseling sessions were not significantly associated with this outcome (all *p* > 0.05) (Table 1).

**TABLE 1:** Univariate logistic regression analysis of factors associated with the use of GnRH agonists for ovarian protection.

Variable	OR (95% CI)	*p*
University hospital	7.74 (3.31–19.13)	< 0.001
Full‐time “oncofertility navigator” present	4.11 (1.57–12.92)	0.01
Awareness of 2027 revision of facility certification criteria in Japan	3.04 (0.80–19.98)	0.16
Awareness of the JSCO Guidelines 2024	3.44 (1.31–10.83)	0.02
AMH and reproductive hormone measurement before and/or after chemotherapy	2.30 (0.91–6.65)	0.10
Annual fertility preservation counseling sessions	1.01 (1.00–1.02)	0.12

*Note:* ORs represent the odds ratio for having experience in the use of GnRH agonists for ovarian protection. ORs for counseling sessions represent the odds ratio per one additional annual session. JSCO Guidelines 2024, Clinical Practice Guidelines for Fertility Preservation in Childhood, Adolescent and Young Adult Cancer Patients 2024.

Subsequently, a multivariable logistic regression analysis was performed, including variables that were significant in the univariate analyses as well as a clinically important indicator of institutional reproductive medicine practice, the measurement of AMH and reproductive hormones before and/or after chemotherapy. The interaction term between university hospital status and presence of a full‐time “oncofertility navigator” was tested, but no significant interaction was found (*p* = 0.99). Therefore, both variables were included as independent covariates in the final multivariate model. In the multivariate logistic regression analysis, being a university hospital (adjusted OR 7.57, 95% CI 3.00–20.63, *p* < 0.001) and the presence of a full‐time “oncofertility navigator” (adjusted OR 3.88, 95% CI 1.30–13.62, *p* = 0.02) remained independently and significantly associated with experience in the use of GnRH agonists for ovarian protection. In contrast, awareness of the JSCO Guidelines 2024 and measurement of AMH and reproductive hormones before and/or after chemotherapy were not significantly associated with this outcome (all *p* > 0.05) (Table 2).

**TABLE 2:** Multivariate logistic regression analysis of factors associated with the use of GnRH agonists for ovarian protection.

Variable	Adjusted OR (95% CI)	*p*
University hospital	7.57 (3.00–20.63)	< 0.001
Full‐time “oncofertility navigator” present	3.88 (1.30–13.62)	0.02
Awareness of the JSCO Guidelines 2024	1.8 (0.57–6.36)	0.33
AMH and reproductive hormone measurement before and after chemotherapy	0.86 (0.27–2.89)	0.81

*Note:* Adjusted odds ratios (ORs) represent the likelihood of having experience in the use of GnRH agonists for ovarian protection after adjusting for all variables shown.

### 3.3 Experience With GnRH Antagonists

Only 5 of the 128 institutions (3.9%) reported experience prescribing GnRH antagonists for ovarian protection within their own institutions. In addition, only 5 of the 128 institutions reported having encountered cases in which GnRH antagonists were used for ovarian protection at other institutions. Among these limited reports, the malignancies included breast cancer, hematologic malignancies, and gynecologic malignancies (Table S2). The reasons reported for selecting GnRH antagonists included limited time before chemotherapy initiation and contraindications to GnRH agonist injections due to thrombocytopenia or coagulation disorders.

### 3.4 Future Perspectives on GnRH Analogs Use

Of the 128 facilities, 86 (67.2%) indicated that they would be more likely to prescribe GnRH analogs for ovarian protection if these treatments were covered by the national insurance system. However, several respondents expressed concern about potential overuse in cases where such treatment might not be necessary.

## 4 Discussion

This nationwide survey is the first to identify institutional factors associated with the clinical use of GnRH analogs for ovarian protection in patients receiving chemotherapy in Japan. Although awareness of GnRH agonist use was high, actual prescription remained limited, with only one in four facilities having experience prescribing them. University hospital status and the presence of a full‐time “oncofertility navigator” were independently associated with GnRH agonist use, suggesting that institutional infrastructure and specialized personnel play key roles in translating awareness into practice. In contrast, although only a small number of institutions reported direct experience prescribing GnRH antagonists within their own institutions, only a small number of institutions reported having encountered cases in which GnRH antagonists were used at other facilities. These agents were therefore selected only in highly specific clinical situations, such as when the time available before initiating chemotherapy was limited or when the use of GnRH agonists was difficult. Overall, these findings reveal that, while awareness of GnRH agonists was high, the clinical implementation of ovarian protection strategies remains insufficient in Japan.

Although the efficacy of GnRH agonists for ovarian protection has been reported in international clinical trials such as the POEMS/SWOG S0230 and PROMISE‐GIM6 studies, including reductions in the incidence of chemotherapy‐induced POI and higher rates of menstrual resumption and subsequent pregnancy, these findings have not necessarily translated into widespread clinical implementation [14, 15, 16]. According to the ESMO and ASCO Clinical Practice Guidelines, GnRH agonists are not positioned as a standard substitute for established fertility preservation methods—such as oocyte, embryo, or ovarian tissue cryopreservation—but rather as a complementary or adjunctive option to the standard fertility preservation method for selected malignancies such as breast cancer [17, 18]. In Japan, the use of GnRH agonists for ovarian protection in patients with selected malignancies, including breast cancer, is also conditionally recommended, but only as an alternative when fertility preservation is not pursued, partly because this indication is not covered by the national health insurance system. Consistent with this positioning, our findings indicate that clinical implementation at the national level remains limited [7, 13].

With regard to GnRH antagonists, preclinical animal studies have suggested potential ovarian‐protective effects through the rapid suppression of pituitary gonadotropin secretion; however, their clinical utility in humans has been minimally investigated to date [8, 9, 10]. In the present survey, GnRH antagonists were selected only in specific clinical situations, such as when chemotherapy needed to be initiated urgently or when GnRH agonist injections were contraindicated because of thrombocytopenia or coagulation disorders.

Taken together, although GnRH agonists are supported by biological and theoretical rationales for ovarian protection, the available clinical evidence remains limited, and they are positioned as adjunctive or conditional options for fertility preservation in many countries, including Japan. In contrast, the clinical role of GnRH antagonists remains exploratory, with very limited real‐world experience. The finding that many institutions reported a greater likelihood of use if public insurance coverage were available suggests that the clinical implementation of GnRH analogs is strongly influenced by institutional and systemic factors. As a result, opportunities to accumulate clinical experience and data necessary for the comprehensive evaluation of their efficacy and limitations may be restricted. Previous surveys have similarly reported substantial variability in fertility preservation practices across institutions and countries, reflecting differences in available expertise, institutional infrastructure, and clinical resources [19, 20, 21]. Future efforts to establish clinical environments in which GnRH analogs can be appropriately evaluated may facilitate the accumulation of evidence regarding their efficacy and safety in ovarian protection.

The “oncofertility navigator” is a certified professional role unique to Japan, established through a nationwide training program and grounded in practical experience in oncofertility care [22, 23]. “Oncofertility navigators” not only coordinate fertility preservation procedures but also facilitate communication between oncology and reproductive teams, ensuring timely referrals and patient counseling. In the present study, an association was observed between the presence of a full‐time “oncofertility navigator” and the use of GnRH agonists for ovarian protection. However, given the cross‐sectional design of this study, a causal relationship cannot be inferred. Although potential confounding factors were considered and included in the multivariable analysis, institutions with full‐time “oncofertility navigators” may already be more actively engaged in oncofertility care. Accordingly, the presence of dedicated personnel may reflect broader institutional infrastructure that supports the translation of awareness into clinical practice, rather than representing an independent causal factor. Further studies are warranted to clarify the specific role of “oncofertility navigators” within institutional oncofertility systems.

The strength of this study lies in being the first large‐scale nationwide survey targeting institutions providing fertility preservation in Japan, thereby elucidating the current status of ovarian protection practices. In addition to identifying the experience of GnRH agonist prescription, this study also evaluated physicians' awareness and future intentions, highlighting institutional and educational factors influencing clinical implementation. Moreover, this study investigated the use of GnRH antagonists, which has rarely been reported, and identified the clinical situations in which they were selected.

There are, however, several limitations to this study that should be acknowledged. First, because this study relied on self‐reported data from a questionnaire survey, reporting bias cannot be ruled out. The responses from nonparticipating institutions were not captured; therefore, the findings may not fully represent all facilities nationwide. Second, the number of facilities that had used GnRH antagonists was small, limiting the ability to perform detailed subgroup analyses or robust statistical comparisons. Third, the cross‐sectional design of this study precludes causal inference regarding institutional factors associated with GnRH analog use. Finally, this survey targeted fertility preservation facilities accredited by JSOG, and the responses were primarily obtained from obstetricians and gynecologists. As a result, cases in which GnRH analogs were independently prescribed in other departments—such as orthopedics, hematology, pediatrics, or breast oncology—were not captured. Therefore, the findings may not directly represent the use of GnRH analogs across all medical specialties nationwide. In addition, this study did not evaluate whether GnRH analogs were used alone or in combination with specific fertility preservation methods, such as oocyte, embryo, or ovarian tissue cryopreservation.

In conclusion, this nationwide study clarified the current status of GnRH analog use for ovarian protection in Japan. Although awareness of recommendations for GnRH agonists was high, actual clinical implementation remained limited, indicating a gap between current clinical practice and international evidence. In particular, many institutions identified the availability of public insurance coverage as an important factor influencing clinical decision‐making, suggesting that institutional and policy‐related factors may affect the implementation of GnRH analog use. Future considerations should involve the careful evaluation of the role of GnRH analogs in fertility preservation, based on the accumulation of clinical data regarding their efficacy and safety, as well as thoughtful discussion of their positioning within existing clinical frameworks and healthcare systems.

## Author Contributions

**Shina Sakaguchi:** software, data curation, investigation, validation, formal analysis, visualization, writing – original draft. **Eishin Nakamura:** methodology, software, data curation, investigation, formal analysis, funding acquisition, resources, writing – review and editing. **Haipeng Huang:** study conception and design, overall project planning, and interpretation of the results. **Yasushi Takai:** conceptualization, methodology, validation, supervision, project administration, resources, writing – review and editing.

## Funding

This work was supported by Ministry of Health, Labour and Welfare (23EA1016).

## Disclosure

An earlier version of this article was presented at the 5th Asian Society of Fertility Preservation (ASFP) Congress, held in Malaysia on August 1, 2025.

## Ethics Statement

This study was approved by the Ethics Committee of Saitama Medical Center, Saitama Medical University (approval number: 2024‐232).

## Consent

No written informed consent was obtained because this study did not include any patient‐identifiable data.

## Conflicts of Interest

The authors declare no conflicts of interest or financial disclosures relevant to the content of this manuscript. Dr. Yasushi Takai is an Editorial Board member of the *Journal of Obstetrics and Gynecology Research* and a co‐author of this article. To minimize bias, he was excluded from all editorial decision‐making related to the acceptance of this manuscript.

## Supporting information


**Table S1:** English version of the questionnaire used in this nationwide survey.
**Table S2:**. Summary of selected questionnaire responses not presented in the main figures.

## Data Availability

The data underlying this study consist of facility‐level responses collected through a nationwide questionnaire survey. These data are not publicly available due to ethical restrictions and the potential risk of identifying participating institutions, even after anonymization. The study protocol was approved by the institutional review board. De‐identified data may be made available for academic purposes upon reasonable request to the corresponding author, subject to institutional approval.

## References

[1] D. Meirow , H. Biederman , R. A. Anderson , and W. H. Wallace , “Toxicity of Chemotherapy and Radiation on Female Reproduction,” Clinical Obstetrics and Gynecology 53 (2010): 727–739. 21048440 10.1097/GRF.0b013e3181f96b54

[2] T. H. M. Keegan , R. Abrahão , and E. M. Alvarez , “Survival Trends Among Adolescents and Young Adults Diagnosed With Cancer in the United States: Comparisons With Children and Older Adults,” Journal of Clinical Oncology 42 (2024): 630–641. 37883740 10.1200/JCO.23.01367 PMC12040215

[3] L. Chen , Z. Dong , and X. Chen , “Fertility Preservation in Pediatric Healthcare: A Review,” Frontiers in Endocrinology (Lausanne) 14 (2023): 1147898. 10.3389/fendo.2023.1147898 PMC10189781 37206440

[4] M. Harada , F. Kimura , Y. Takai , et al., “Japan Society of Clinical Oncology Clinical Practice Guidelines 2017 for Fertility Preservation in Childhood, Adolescent, and Young Adult Cancer Patients: Part 1,” International Journal of Clinical Oncology 27 (2022): 265–280. 34973107 10.1007/s10147-021-02081-w PMC8816532

[5] Z. Blumenfeld , “Fertility Preservation Using GnRH Agonists: Rationale, Possible Mechanisms, and Explanation of Controversy,” Clinical Medicine Insights. Reproductive Health 13 (2019): 1179558119870163. 31488958 10.1177/1179558119870163 PMC6710670

[6] F. Poggio , M. Lambertini , C. Bighin , et al., “Potential Mechanisms of Ovarian Protection With Gonadotropin‐Releasing Hormone Agonist in Breast Cancer Patients: A Review,” Clinical Medicine Insights: Reproductive Health 13 (2019): 1179558119864584. 31391786 10.1177/1179558119864584 PMC6669835

[7] A. Tozawa , F. Kimura , Y. Takai , et al., “Japan Society of Clinical Oncology Clinical Practice Guidelines 2017 for Fertility Preservation in Childhood, Adolescent, and Young Adult Cancer Patients: Part 2,” International Journal of Clinical Oncology 27 (2022): 281–300. 35022887 10.1007/s10147-021-02076-7 PMC8827301

[8] C. N. Lemos , F. M. Reis , G. N. Pena , L. C. Silveira , and A. F. Camargos , “Assessment of Fertility Protection and Ovarian Reserve With GnRH Antagonist in Rats Undergoing Chemotherapy With Cyclophosphamide,” Reproductive Biology and Endocrinology 8 (2010): 51. 20482803 10.1186/1477-7827-8-51 PMC2885402

[9] X. Li , X. Kang , Q. Deng , J. Cai , and Z. Wang , “Combination of a GnRH Agonist With an Antagonist Prevents Flare‐Up Effects and Protects Primordial Ovarian Follicles in the Rat Ovary From Cisplatin‐Induced Toxicity: A Controlled Experimental Animal Study,” Reproductive Biology and Endocrinology 11 (2013): 16. 23452939 10.1186/1477-7827-11-16 PMC3598983

[10] M. Tas , G. Oner , P. Ulug , A. Yavuz , and B. Ozcelik , “Evaluation of Protective Effects of GnRH Agonist or Antagonist on Ovarian Reserve With Anti‐Müllerian Hormone and Histological Analysis in a Rat Model Using Cisplatin,” Archives of Medical Science 19 (2023): 448–451. 37034528 10.5114/aoms.2019.87540 PMC10074176

[11] C. Kunitomi , M. Harada , Y. Sanada , et al., “The Possible Effects of the Japan Society of Clinical Oncology Clinical Practice Guidelines 2017 on the Practice of Fertility Preservation in Female Cancer Patients in Japan,” Reproductive Medicine and Biology 21 (2022): e12453. 35386371 10.1002/rmb2.12453 PMC8967277

[12] M. Imamura , K. Hirata , M. Unno , et al., “Current Status of Projects for Developing Cancer‐Related Clinical Practice Guidelines in Japan and Recommendations for the Future,” International Journal of Clinical Oncology 24 (2019): 189–195. 30143906 10.1007/s10147-018-1340-1 PMC6373226

[13] Japan Society of Clinical Oncology , Clinical Practice Guidelines for Fertility Preservation in Pediatric, Adolescent, and Young Adult Cancer Patients — 2nd Edition (Kanehara & Co., Ltd, 2024).

[14] L. Del Mastro , L. Boni , A. Michelotti , et al., “Effect of the Gonadotropin‐Releasing Hormone Analogue Triptorelin on the Occurrence of Chemotherapy‐Induced Early Menopause in Premenopausal Women With Breast Cancer: A Randomized Trial,” JAMA 306 (2011): 269–276. 21771987 10.1001/jama.2011.991

[15] H. C. Moore , J. M. Unger , K. A. Phillips , et al., “Goserelin for Ovarian Protection During Breast‐Cancer Adjuvant Chemotherapy,” New England Journal of Medicine 372 (2015): 923–932. 25738668 10.1056/NEJMoa1413204 PMC4405231

[16] H. C. F. Moore , J. M. Unger , K. A. Phillips , et al., “Final Analysis of the Prevention of Early Menopause Study (POEMS)/SWOG Intergroup S0230,” Journal of the National Cancer Institute 111 (2019): 210–213. 30371800 10.1093/jnci/djy185 PMC6657277

[17] M. Lambertini , F. A. Peccatori , I. Demeestere , et al., “Fertility Preservation and Post‐Treatment Pregnancies in Post‐Pubertal Cancer Patients: ESMO Clinical Practice Guidelines(†),” Annals of Oncology 31 (2020): 1664–1678. 32976936 10.1016/j.annonc.2020.09.006

[18] H. I. Su , C. Lacchetti , J. Letourneau , et al., “Erratum: Fertility Preservation in People With Cancer: ASCO Guideline Update,” Journal of Clinical Oncology 43 (2025): 2553. 10.1200/JCO-25-01373 40554739

[19] A. S. Rashedi , S. F. de Roo , L. M. Ataman , et al., “Survey of Fertility Preservation Options Available to Patients With Cancer Around the Globe,” JCO Global Oncology 6 (2020): 331–344. 10.1200/JGO.2016.008144 PMC7853877 32259160

[20] N. Ozimek , M. Salama , and T. K. Woodruff , “National Oncofertility Registries Around the Globe: A Pilot Survey,” Frontiers in Endocrinology (Lausanne) 14 (2023): 1148314. 10.3389/fendo.2023.1148314 PMC10200897 37223027

[21] S. Y. Baek , K. H. Lee , S. B. Kim , et al., “Knowledge, Attitudes, and Behaviors Toward Fertility Preservation in Patients With Breast Cancer: A Cross‐Sectional Survey of Physicians,” Frontiers in Oncology 13 (2023): 1109694. 36756160 10.3389/fonc.2023.1109694 PMC9899882

[22] M. Ono , M. Harada , A. Horie , et al., “Effect of a Web‐Based Fertility Preservation Training Program for Medical Professionals in Japan,” International Journal of Clinical Oncology 28 (2023): 1112–1120. 37322221 10.1007/s10147-023-02366-2

[23] Y. Takai , “Recent Advances in Oncofertility Care Worldwide and in Japan,” Reproductive Medicine and Biology 17 (2018): 356–368. 30377391 10.1002/rmb2.12214 PMC6194250

