# Supplementary material for: Nationwide Survey of Institutional Factors Related to the Use of Gonadotropin‐Releasing Hormone Analogs for Ovarian Protection in Women Receiving Chemotherapy in Japan
Source: J Obstet Gynaecol Res. 2026 Apr 6;52(4):e70273. doi: 10.1111/jog.70273 (PMC13054117; doi:10.1111/jog.70273)
Supplement: Supplementary file 1 — Table S1: English version of the questionnaire used in this nationwide survey. Table S2:. Summary of selected questionnaire responses not presented in the main figures. [file JOG-52-0-s001.docx]

**Supplementary Table S1. English version of the questionnaire used in this nationwide survey**

**Section 1. About participation**

Q1. Consent to participate in this questionnaire survey

Agree / Disagree

**Section 2. Facility information**

Q2. Type of institution

University hospital / National or municipal hospital / Private hospital / Private clinic / Others (please specify):

Q3. Approximate number of fertility preservation counseling sessions per year (both male and female patients; approximate number acceptable)

0–5 / 6–10 / 11–20 / 21–50 / 51–100 / 101–200 / >200

Q4. Is a certified oncofertility navigator working full time at your facility?

Yes / No

**Section 3. Regarding GnRH agonists**

Q5. Are you aware that GnRH agonists are recommended for ovarian protection in certain cancers (e.g., breast cancer)?

Yes / No

Q6. Has your facility used GnRH agonists for ovarian protection?

Yes (Proceed to Q7) / No (Skip to Q9)

Q7. Cost category for GnRH agonist use

Out-of-pocket / Covered by insurance / Case-dependent / Others (please specify):

Q8. Are serum AMH or other hormone levels measured before and/or after GnRH agonist administration?

Yes / No / Others (please specify):

Q9. Have you encountered cases in which GnRH agonists were used for ovarian protection at other institutions?

Yes / No

Q10. Cancers for which GnRH agonists were used for ovarian protection (select all that apply)

Breast, Hematologic, Gynecologic, Collagen disease, Pediatric, Bone/soft tissue, Gastrointestinal, Brain, Lung, Head and neck, Urologic, Others

**Section 4. Regarding GnRH antagonists**

Q11. Has your facility used GnRH antagonists for ovarian protection?

Yes (Proceed to Q12) / No (Skip to Q15)

Q12. Cost category for GnRH antagonist use

Out-of-pocket / Covered by insurance / Case-dependent / Others (please specify):

Q13. Are serum AMH or other hormone levels measured before and/or after GnRH antagonist administration?

Yes / No

Q14. Reasons for selecting GnRH antagonist (select all that apply)

Short interval before starting chemotherapy / Contraindication to subcutaneous injection (e.g., thrombocytopenia, coagulopathy) / Others (please specify):

Q15. Have you encountered cases in which GnRH antagonists were used for ovarian protection at other institutions?

Yes / No

Q16. Cancers for which GnRH antagonists were used for ovarian protection (select all that apply)

Breast, Hematologic, Gynecologic, Collagen disease, Pediatric, Bone/soft tissue, Gastrointestinal, Brain, Lung, Head and neck, Urologic, Others

**Section 5. Awareness and perception**

Q17. Are you aware that, from 2027, fertility preservation facilities will be required to conduct ≥5 counseling cases per year and to have a full-time certified navigator?

Yes / No

Q18. Are you aware of or do you possess the 2024 revised edition of the JSCO Fertility Preservation Guidelines?

Have / Know but not have / Do not know

Q19. If GnRH agonists/antagonists are approved for insurance reimbursement for ovarian protection, do you think prescription opportunities will increase?

Increase / No change / Others (please specify):

Note: This questionnaire was originally created in Japanese using Google Forms (Google LLC, Mountain View, CA, USA). The English version is provided for reference.

**Supplementary Table S2. Summary of selected questionnaire responses not presented in the main figures**

| **Response category** |  | **n (%)** |
| --- | --- | --- |
| **Q7. Cost of GnRH agonists for ovarian protection (n = 34)** | | |
| Out-of-pocket |  | 10 (29.4) |
| Insurance |  | 7 (20.6) |
| Case-dependent |  | 15 (44.1) |
| Uncertain |  | 2 (5.9) |
| **Q9. Encountered cases in which GnRH agonists were used for ovarian protection at other institutions (n = 128)** | | |
| Yes |  | 107 (83.6) |
| No |  | 21 (16.4) |
| **Q11. Experience using GnRH antagonists for ovarian protection (n = 128)** | | |
| Yes |  | 5 (3.9) |
| No |  | 123 (96.1) |
| **Q12. Encountered cases in which GnRH antagonists were used for ovarian protection at other institutions (n = 128)** | | |
| Yes |  | 5 (3.9) |
| No |  | 114 (89.1) |
| Unknown |  | 9 (7.0) |
| **Q14. Reasons for selecting GnRH antagonists (multiple responses allowed, n = 5)** | | |
| Short interval before starting chemotherapy | | 4 (80.0) |
| Contraindication to subcutaneous injection (e.g., thrombocytopenia, coagulopathy) | | 3 (60.0) |
| **Q16. Cancers for which GnRH antagonists were used for ovarian protection (multiple responses allowed, n = 104)** | | |
| Breast cancer |  | 18 (17.3) |
| Hematologic malignancies |  | 12 (11.5) |
| Gynecologic malignancies |  | 10 (9.6) |
| Collagen disease |  | 2 (1.9) |
| Pediatric malignancies |  | 2 (1.8) |
| Bone/soft tissue tumors |  | 1 (1.0) |
| Gastrointestinal cancer |  | 1 (1.0) |
| Brain tumor |  | 2 (1.9) |
| Lung cancer |  | 2 (1.9) |
| Head and neck cancer |  | 1 (1.0) |
| Urologic cancer |  | 1 (1.0) |
| Others / none / unknown |  | 76 (73.1) |
| **Q18. Awareness or possession of the 2024 revised JSCO Fertility Preservation Guidelines (n = 128)** | | |
| Have |  | 88 (68.8) |
| Know but do not have |  | 29 (22.7) |
| Do not know |  | 11 (8.6) |
| **Q19. Expected change in prescription if GnRH agonists/antagonists are approved for insurance reimbursement (n = 128)** | | |
| Increase |  | 86 (67.2) |
| No change |  | 23 (18.0) |
| Do not know |  | 18 (14.1) |
| Others |  | 1 (0.8) |

Percentages may not total 100% due to rounding. Multiple responses were allowed for selected questionnaire items. For items with a limited number of respondents, the denominator is indicated for each question.
